# Predicting Productive Performance in Grow-Finisher Pigs Using Birth and Weaning Body Weight

**DOI:** 10.3390/ani10061017

**Published:** 2020-06-12

**Authors:** Jordi Camp Montoro, Edgar Garcia Manzanilla, David Solà-Oriol, Ramon Muns, Josep Gasa, Oliver Clear, Julia Adriana Calderón Díaz

**Affiliations:** 1 Pig Development Department, Animal and Grassland Research and Innovation Centre, Teagasc, Moorepark, Fermoy, Co. Cork, Cork P61 C996, Ireland; egmanzanilla@gmail.com (E.G.M.); oliverclear78@gmail.com (O.C.); Julia.CalderonDiaz@teagasc.ie (J.A.C.D.); 2 Department of Animal and Food Sciences, Animal Nutrition and Welfare Service, Universitat Autònoma de Barcelona, 08193 Bellaterra, Spain; David.Sola@uab.cat (D.S.-O.); Josep.Gasa@uab.cat (J.G.); 3 UCD Veterinary Sciences Centre, University College Dublin, Belfield, Dublin 4, D04 V1W8, Ireland; 4 Agri-Food and Biosciences Institute, Large Park, Hillsborough, Co Down BT 26 6DR, Northern Ireland, UK; Ramon.Muns@afbini.gov.uk

**Keywords:** body weight variability, growth performance, pig, regression tree, receiver operating characteristic curve, slow growing pigs, swine

## Abstract

**Simple Summary:**

Smaller than average sized pigs requiring extra time to reach target slaughter weight are often referred to as slow growing pigs. This subset of pigs poses a management challenge and may have economic implications for pig producers. The aims of this study were to investigate the effect of birth and weaning body weight on performance of grow-finisher pigs and to estimate cut-off values for birth and weaning body weight in order to identify slow growing pigs early in life. Pigs with low birth and weaning weight grew slower compared to pigs with higher birth or weaning weight, although feed conversion efficiency was similar for both groups. Pigs weaned at approximately 28 days weighing less than 3.7 kg would be identified as slow growing pigs within a batch. Moreover, a cut-off value of 1.1 kg of body weight at birth and 6.4 kg of body weight at weaning would allow pig producers to identify the pigs that are likely to reach target slaughter weight at 22 weeks of age. In conclusion, birth body weight does not always determine subsequent growth performance, and the cut-off values identified could be used on farm to design new management and nutritional strategies for slow growing pigs.

**Abstract:**

This study aimed to (1) investigate the effect of birth and weaning body weight (BW) on performance indicators of grow-finisher pigs and (2) estimate birth and weaning BW cut-off values in order to identify slow growing pigs (SGP). Pigs (n = 144) were classified as SMALL (0.9 ± 0.13 kg) or BIG (1.4 ± 0.20 kg) at birth and re-classified as SMALL (5.4 ± 1.6 kg) or BIG (6.3 ± 1.91 kg) at weaning. Individual BW was recorded bi-weekly, and feed intake was recorded on a daily basis. Average daily gain (ADG) and feed intake, feed conversion ratio (FCR) and days to target slaughter weight (TSW) were calculated. SMALL–SMALL pigs had lower ADG (*p* < 0.05) requiring 167.1 days (i.e., 14.2 extra days) to TSW (*p* < 0.05) compared with BIG pigs at birth and/or weaning. However, FCR was similar between groups (*p* > 0.05). Pigs weaned at <3.7 kg BW would likely be SGP. Pigs born at ≥1.1 kg BW or weaned at ≥6.4 kg BW are more likely to reach TSW at 22 weeks of age. The results suggest that birth BW might not be the best predictor for subsequent performance, as some small-born pigs were able to catch up with their bigger counterparts. The cut-off values identified could be used to design specific management and nutritional strategies for SGP.

## 1. Introduction

Improving production efficiency during the grow-finisher stage is crucial. This stage is the most expensive period in pig production, accounting for over 65% of the total cost of production [1], and minor improvements result in important increases in profit for farmers. One of the main factors impacting the production efficiency is increased body weight (BW) variability. Additionally, birth and weaning BW are among the most critical factors for lifetime growth performance in pigs [2,3,4]. The continuous genetic advancement over the last decade has increased litter size at birth, leading to a considerable decrease in average birth weight, increased percentage of piglets born with light weight [5,6] and increased BW variability at birth [7]. Piglets that are born small often remain stunted and are unable to catch up to their big counterparts during the entire production cycle [5,6,8]. Some studies have established ≤1.25 kg as low birth BW [2,4,9], while recent work suggests that 15 to 25% of new-born piglets are born under 1.1 kg of BW [10]. Moreover, Quiniou et al. [5] stated that the proportion of piglets weighing less than 1 kg at birth increases from 7 to 23% in large litters (≥16 piglets per sow). Similarly, several studies also indicated weaning BW as a critical factor for post-weaning growth and time to reach target slaughter weight [3,11,12].

Light birth and/or weaning BW pigs that are in the lower quartile of the BW population distribution and require extra time to reach target slaughter weight are often referred to as slow growing pigs (SGP) [2,4]. It is estimated that, in a given batch, 10 to 15% of pigs are SGP [4,13,14]. These pigs are susceptible to higher mortality rates throughout the production cycle [15,16,17], and those that survive pose management challenges in all-in-all-out production systems [18]. For instance, contrary to all-in-all-out principles, farmers may hold back SGP to a similarly sized following batch of younger animals to facilitate management [13]. Calderón Díaz et al. [13] reported that carcasses from pigs repeatedly delayed during the production cycle were 10 kg lighter than pigs that were not delayed. In addition, such practice increases the likelihood of disease spread and occurrence [13] and increases the occupation time of the facilities. This leads to increased feed costs and the production of fewer finisher pigs per pig space per year. Additionally, carcasses from SGP are more likely to have poor grading due to higher fat content as a result of the extended time they require to reach target slaughter weight [4], thus decreasing the efficiency of the whole production cycle [2,19].

Knowing SGP key performance indicators, such as average daily gain (ADG), average daily feed intake (ADFI), feed conversion ratio (FCR) and time needed to reach target slaughter weight, would allow farmers to improve the management of the whole herd. There is conflicting information in the scientific literature regarding SGP key performance indicators [2,20]. Some authors reported that light weight pigs at birth and/or at weaning have poor growth performance compared to their heavier counterparts [4,5,21], while other authors reported that light birth BW pigs are able to catch up their bigger counterparts and to have a similar BW by the end of the production cycle [22,23,24]. However, previous research has not estimated cut-off values for birth and/or weaning BW to differentiate between pigs that are born small but are able to catch up with their big counterparts and those that remain small for the whole production cycle. By identifying SGP earlier in life, farmers could design new management and nutritional strategies to improve SGP growth performance, improving farm production efficiency.

We hypothesize that pigs with low BW at birth and at weaning would have decreased growth performance in the whole grow-finisher period compared with their heavier counterparts at birth or at weaning. We hypothesize that SGP will be identified by having cut-off values for birth and weaning weight lower than the population mean. Thus, the objectives of this study were to (1) investigate the effect of birth and weaning BW on key performance indicators of grow-finisher pigs and (2) identify cut-off values for birth and weaning BW in order to recognize SGP early in life.

## 2. Materials and Methods

### 2.1. Care and Use of Animals

The study received ethical approval from the Teagasc Animal Ethics Committee (TAEC 184/2018), and it was conducted at the Teagasc Pig Research Unit in Fermoy, Co. Cork, Ireland. A total of 370 pigs [194 females and 176 males; Danish Duroc × (Large White × Landrace)] born within one week were individually ear-tagged, and their BW was recorded within 24 h after birth. Information on sow parity and litter size was also recorded. Pigs were classified according to their birth BW as small (SMALL; 0.9 ± 0.23 kg) if BW ≤ 1.15 kg, or big (BIG; 1.4 ± 0.20 kg) if BW > 1.15 kg. The 1.15 kg BW cut-off value was selected considering previous literature research [4,9,10] and the lower quartile of the birth BW population distribution from the batch of pigs used for this study. Pigs were weaned at approximately 28 days, individually weighed and re-classified as SMALL (4.3 ± 1.11 kg) if BW ≤ 5.5 kg or BIG (7.4 ± 1.86 kg) if BW > 5.5 kg, yielding a 2 × 2 factorial arrangement. The 5.5 kg BW cut-off value at weaning was selected considering the lower quartile of the weaning BW population distribution from the batch of pigs used for this study. Pigs were matched according to sow parity and litter size, and 144 pigs (64 males and 80 females) were selected. Pigs were classified into one of four groups (n = 36 pigs/group): SMALL–SMALL (15 males and 21 females), SMALL–BIG (15 males and 21 females), BIG–SMALL (17 males and 19 females) and BIG–BIG (16 males and 20 females). Descriptive statistics by group are provided in Table 1. At weaning, pigs were fitted with a transponder and transferred to the nursery stage accommodation. Pigs were housed in mixed sex pens (n = 12 pigs per pen; 0.55 m^2^ per pig) with fully slatted plastic floor equipped with individual feeding stations (MLP-ECO, ASR 500, Schauer, Prambachkirchen, Austria) to record individual daily feed intake. Pigs underwent a training period of 15 days to get habituated to use the feeding stations as per normal practice at the Teagasc Pig Research Facility. During this period, feeding stations were switched off and feed bins were manually filled, and thus, feed intake was not recorded. Pigs received a common starter diet [20.0% crude protein (CP), 12.34 MJ of net energy (NE) and 1.40% standard ileal digestible (SID) lysine per kg of feed] for seven days, link diet (19.0% CP, 10.96 MJ/NE and 1.28% SID lysine per kg of feed) for 18 days and soybean meal–barley–wheat based nursery diet (17.75% CP, 10.63 MJ/NE and 1.04% SID lysine per kg of feed) for 28 days. At 53 days post-weaning, pigs were transferred to the finisher accommodation, where they remained until reaching the target slaughter weight, which was at least 110 kg of BW as per normal practice in Irish pig farms. During the finisher stage, pigs were housed in the same mixed sex groups with a minimum space of 0.95 m^2^ per pig in pens with fully slatted concrete floor equipped with individual feeding stations (MLP-ECO, ASR 500, Schauer, Prambachkirchen, Austria). Pigs were fed ad libitum a common soybean meal–maize–wheat based finisher diet (16.18% CP, 9.67 MJ/NE and 0.92% SID lysine per kg of feed). In the nursery and finisher stages, the temperature was controlled by a mechanical ventilation system with fan speed and air inlet area regulated by a climate controller.

### 2.2. Measurements

Pigs were individually weighed using a digital scale (R323, Rinstrum, Langenfeld, Germany) every two weeks starting at 16 days post-weaning until they reached target slaughter weight (i.e., at least 110 kg). Average daily gain was calculated for every two-weeks interval. Feed intake (FI) was recorded on a daily basis, and ADFI was calculated for every two-week period. For each two-week period, FCR was calculated as follows:

(1) $$FCR=\frac{kg\;of\;feed\;consumed}{kg\;of\;BW\;gain}$$

Additionally, the days to target slaughter weight (DTSW) were recorded.

### 2.3. Statistical Analyses

#### 2.3.1. Body Weight, Feed Intake and Feed Efficiency Traits

Each pig was considered as the experimental unit for all data analyses. Residuals were tested for normality using the Shapiro–Wilk test and by examining the normal probability plot using the UNIVARIATE procedure of SAS v9.4 [25]. Two different analyses were performed: *Model 1* included data from 16 days post-weaning up to 20 weeks of age, when the first group of pigs reached 110 kg of BW and were sent to slaughter. Predicted variables included BW, ADG, FI, ADFI and FCR. Mixed model equation methods accounting for repeated measurements were used in PROC MIXED of SAS v9.4 [25]. *Model 2* included data from 15 days post-weaning until all pigs reached target slaughter weight, and the same predicted variables were investigated as per Model 1 plus DTSW. Data were also analyzed using mixed model equation methods in PROC MIXED of SAS v9.4 [25]. For both analyses, models included birth and weaning body weight classification, observation day and their interaction, and sex as fixed effects. Pig was included as a random effect. Multiple means comparisons were done using Tukey–Kramer’s correction. Results for the fixed effects are reported as least square means ± standard error. Alpha level for determination of significance and trends were 0.05 and 0.10, respectively.

#### 2.3.2. Birth and Weaning Body Weight Cut-Off Values

Two different analyses were used to estimate cut-off values for birth and weaning BW to identify SGP, namely regression tree analysis and Receiver Operating Characteristic (ROC) Curves analysis. For the regression tree analysis, data were analyzed using the *rpart* package [26] of R v3.5.2 [27]. The model included DTSW as the outcome variable and birth and weaning BW as predictor variables. Then, analysis of variance (ANOVA) was performed using the *stats* package in R v3.5.2 [27] to confirm that the groups created were statistically different from each other. ROC curve analysis was used to estimate cut-off values for birth and weaning BW to identify pigs that would reach target slaughter weight at 22 weeks of age. The age to slaughter was selected based on Irish commercial criteria and the farm performance. First, pigs were dichotomized based on whether or not reaching target slaughter weight at 22 weeks, and data were analyzed using the *pROC* package [28] of R v3.5.2 [27]. Univariable and bi-variable models were used in this analysis. To evaluate the overall performance of the models, sensitivity and specificity were calculated at various cut-off values. Sensitivity was defined as the proportion of pigs correctly classified as reaching target slaughter BW at 22 weeks of age. Specificity was defined as the proportion of pigs correctly classified as not reaching target slaughter BW at 22 weeks of age. The accuracy of the models was assessed by calculating the area under the curve (AUC). Values of AUC were interpreted as non-accurate (AUC = 0.5), less accurate (0.5 < AUC ≤ 0.7), moderately accurate (0.7 < AUC ≤ 0.9), highly accurate (0.9 < AUC < 1) and perfect (AUC = 1) [29]. The Youden Index was used to identify the optimal cut-off value that would separate the sample into two populations [30].

## 3. Results

### 3.1. Body Weight, Feed Intake and Feed Efficiency Traits

#### 3.1.1. Model 1

There was an interaction between birth and weaning BW when predicting BW, ADG, ADFI and FCR. There were no differences for ADG and ADFI between SMALL–BIG, BIG–SMALL and BIG–BIG pigs from 6 to 20 weeks of age (*p* > 0.05; Table 2). Pigs classified as SMALL–SMALL were 15.8 kg lighter than the other groups at 20 weeks of age (*p* < 0.05); they tended to gain 97.4 g less per day (*p* < 0.10), and consumed 337 g less feed per day compared to the other groups from 6 to 20 weeks of age (*p* < 0.001; Table 2). Pigs classified as BIG–SMALL had higher FCR compared to the other groups (*p* < 0.001), and no difference was observed for FCR between SMALL–SMALL, SMALL–BIG and BIG–BIG pigs (*p* > 0.05; Table 2). Male pigs gained 47.8 g more per day (*p* = 0.025) and had lower FCR compared to female pigs (*p* < 0.001). There was no difference in BW and ADFI between male and female pigs at 20 weeks of age (*p* > 0.05).

#### 3.1.2. Model 2

There was an interaction between birth and weaning BW when predicting ADG, ADFI, FCR and DTSW. Pigs classified as SMALL–SMALL gained 79.9 g less per day during the grow-finisher period compared to the other groups (*p* < 0.05; Table 3). Additionally, SMALL–SMALL pigs needed 14.2 days more to reach target slaughter weight than the other groups (*p* < 0.001), and no difference was observed in DTSW among SMALL–BIG, BIG–SMALL and BIG–BIG pigs (*p* > 0.05; Table 3). Pigs classified as BIG–SMALL consumed 191.5 g more per day (*p* < 0.001) and had higher FCR during the grow-finisher period compared to the other groups (*p* < 0.05; Table 3). Male pigs consumed 83.3 g less per day (*p* = 0.015), had lower FCR (*p* < 0.001), and they reached target slaughter weight 4.1 days earlier (*p* = 0.047) than female pigs.

### 3.2. Cut-Off Values for Birth and Weaning Body Weight

#### 3.2.1. Regression Tree Analysis

**Figure 1:**
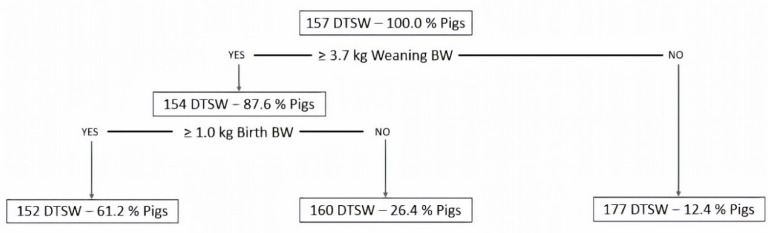
Regression tree analysis used to estimate cut-off values for birth and weaning BW to identify slow growing pigs within a batch. Model included the days to target slaughter weight (DTSW; i.e., 110 kg of BW) as the outcome variable, and birth body weight and weaning body weight as predictor variables. Pigs with body weight lower than 3.7 kg of BW at weaning (i.e., 28 days of age) would be considered slow growing pigs. Regression tree analysis was performed using the *rpart* package [26] of R v3.5.2 [27].

Weaning BW was the main predictor variable to classify pigs based on their age at target slaughter weight. The regression tree classified pigs into three distinctive groups (*p* < 0.001; Figure 1). A first cut-off value was obtained at 3.7 kg of BW at weaning, with pigs with a weaning BW lower than this cut-off value taking 177 days to reach 110 kg of BW and representing 12.4% of pigs (Figure 1). This group of pigs would correspond to SGP. A second cut-off value was observed for pigs with a weaning BW ≥ 3.7 kg, which were further classified into two separate groups based on their birth BW (*p* < 0.05; Figure 1). Pigs with a weaning BW ≥ 3.7 kg and birth BW ≥ 1.0 kg (61.2% of pigs) took 152 days to reach 110 kg of BW, while pigs with a weaning BW ≥ 3.7 kg and birth BW < 1.0 kg (26.4% of pigs) needed eight more days to reach 110 kg of BW (Figure 1).

#### 3.2.2. Receiver Operating Characteristic (ROC) Curve Analysis

**Figure 2:**
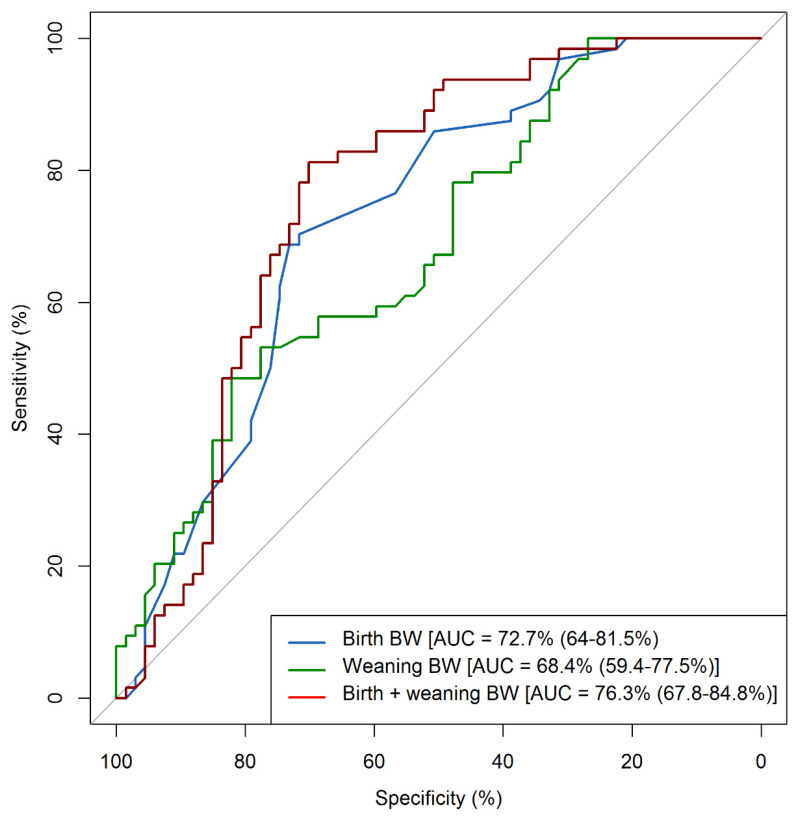
Receiver Operating Characteristic (ROC) curve representing the predictive performance of three different models for identifying pigs that would reach 110 kg of body weight (BW) at 22 weeks of age. Models included birth BW, weaning BW and birth BW + weaning BW as predictor variables. The ROC curve was estimated using the *pROC* package [28] of R v3.5.2 [27]. AUC = area under the curve (95% CI); the diagonal line represents an AUC of 0.5.

Cut-off values for birth and weaning BW were estimated to identify pigs that would reach target slaughter weight at 22 weeks of age. The AUC for the three models ranged from 68.4% (weaning BW as predictor) to 76.3% (birth BW plus weaning BW as predictors), and they were all significantly different from 0.5 (Table 4). The optimal cut-off value for the predictor variables and their associated sensitivity and specificity are also shown in Table 4. When comparing ROCs, no difference was observed between the AUC of the three models (*p* > 0.05). ROCs are shown in Figure 2.

## 4. Discussion

Under the conditions of this study, pigs born and weaned small continued to have decreased growth performance during the grow-finisher period compared with pigs that were classified as big either at birth or at weaning. This result is in line with those of previous studies [4,21,22]. Some light birth weight pigs have fewer muscle fibers [20,31,32] that result in reduced future growth performance, restricting lean growth, increasing fat deposits and resulting in poorer pork quality [20,33,34]. Moreover, light birth weight pigs can have an inadequate colostrum intake due to delaying the first suckle andhave a lower ability to access the best teats and to stimulate them to have higher milk consumption [17,35]. Furthermore, they may show retarded post-weaning maturation of the gastrointestinal tract [36,37], which could contribute to the poorer growth performance observed after weaning in the SMALL–SMALL pigs. On the contrary, SMALL–BIG pigs were able to compensate their light birth weight by having higher ADG during lactation than the average of the batch. Comparisons of the findings with those of other studies [24,38] confirm that this subset of pigs have the potential to grow as fast as heavier pigs at birth during nursery and the grow-finisher period. Previous research pointed out that low birth weight pigs can show various degrees of compensatory growth to finally meet or exceed target slaughter BW of their heavier counterparts [21,39,40]. However, they will only be able to show such compensatory growth if their ADG during lactation is above the average level [23], as we observed with the SMALL–BIG pigs in this study. Explanations to why some piglets can exhibit varying degrees of compensatory growth are reliant on the number of muscle fibers present at birth [39,41] and/or morphometric characteristics at birth [22]. Additionally, one should discriminate between piglets that have been born light for their gestational age and are proportionally small [42] from those that have suffered uterine growth restriction and may remain stunted throughout the production cycle [43].

Pigs with heavier birth weight and lighter weaning weight had similar ADG during lactation to SMALL–SMALL pigs. The causes of this lower growth of the BIG–SMALL pigs were not evaluated since several factors can influence ADG during suckling, such as differences in colostrum and milk production among sows and teats, creep feed intake by the piglets, human handling, as well as health conditions [17,44,45]. However, regardless of the underlying causes, the lower growth of BIG–SMALL pigs shows an interesting reflection on the growth dynamics of piglets during the suckling phase. Nonetheless, in agreement with previous studies [22,23,24], BIG–SMALL pigs showed compensatory growth during the grow-finisher period, achieving a similar body weight to heavier weaning weight pigs by the end of the production cycle.

In Model 1, SMALL–SMALL pigs had lower ADFI compared with the other groups. This result agrees with those observed in earlier studies [46,47]. However, in Model 2, SMALL–SMALL pigs had similar ADFI as SMALL–BIG and BIG–BIG pigs. Daily feed intake increases as pigs get heavier. Thus, for the same age, SMALL–SMALL pigs have different ADFI because they are smaller than the other groups. However, for the same BW, SMALL–SMALL pigs have the same ADFI as their heavier counterparts as a result of spending more time in the facilities to reach target slaughter weight. Interestingly, BIG–SMALL pigs had higher ADFI when they were followed until they reached target slaughter weight, despite their ADFI being similar to SMALL–BIG and BIG–BIG pigs until 20 weeks of age. Pigs classified as BIG–SMALL had higher FCR for the whole grow-finisher period, indicating that they were less efficient in energy utilization although they showed compensatory growth during the grow-finisher stage. This high feed intake but poorer feed efficiency of the BIG–SMALL pigs could be the consequence of a long-term effect caused during the suckling phase. On the contrary, SMALL–SMALL pigs were as feed-efficient as BIG–BIG and SMALL–BIG pigs in both Model 1 and Model 2. This finding is in accordance with previous results reported by Douglas et al. [2], Collins et al. [3] and Paredes et al. [47] but contrary to those reported by Gondret et al. [20], who suggested that the poor growth performance of slow growing pigs was due to poorer feed efficiency. Feed efficiency is a key factor in pork production, with economic and environmental implications. Feed efficiency is affected by many factors, such as diet composition, body composition, feed intake, growth rate, thermal environment, immunological status, feed processing and delivery [48]. Therefore, strategies to improve the growth performance of slow growing pigs could include improving the diet and nutrient composition in the grow-finisher stage [49].

On average, faster growing pigs reached target slaughter weight at 22 weeks of age, while SMALL–SMALL pigs required 14.2 days extra to reach target slaughter weight. This is similar to previous results where slow growing pigs required 10 to 20 days extra to achieve target slaughter weight [4,5,50]. We estimated cut-off values for birth and weaning weight that could be used to identify slow growing pigs early in life. Regression tree analysis was used to calculate a cut-off value for birth and weaning weight considering age at slaughter. Weaning weight appeared to be a better predictor variable than birth weight to classify pigs based on age at slaughter. A cut-off of 3.7 kg of BW at weaning would allow farmers to identify slow growing pigs that would need 23 days extra to achieve target slaughter BW. This finding is consistent with that of Larriestra et al. [15], who established a cut-off value of 3.6 kg of BW at weaning, which maximizes sensitivity and specificity to correctly predict the likelihood of dying or of being light in weight when exiting the nursery stage. Additionally, pigs below 3.6 kg of BW at weaning will require a higher level of management and more complex diets [51]. Using the regression tree analysis, 12.4% of pigs were identified as slow growing pigs. This is in accordance with previous studies that reported 10–15% of slow growing pigs within a batch [4,13,15]. Most farmers weigh pigs at weaning but not at birth, albeit weighing pigs individually is not a common practice in commercial pig farms. Nonetheless, farmers usually sort pigs by size or body weight at weaning. Therefore, the cut-off value of 3.7 kg of BW at weaning could be a valuable indicator for farmers to identify slow growing pigs.

ROC curve method was used to estimate cut-off values for birth and weaning BW to identify pigs that would reach target slaughter weight at 22 weeks of age. A cut-off value of 6.7 kg of BW was obtained using weaning weight as the only predictor variable. This is consistent with previous reports which stated that 80% of pigs weaned at 28 days of age at <6.4 kg of BW became slow growing pigs at slaughter age [4]. Recently, López-Vergé et al. [52] reported 6.88 kg of BW at weaning as a threshold to account for better productive performance in the grow-finisher period. A cut-off value of 1.1 kg of BW was obtained when birth BW was considered as the only predictor variable in the ROC curve analysis. This is similar to previous results showing 1 kg of BW at birth as a critical value for higher risk of mortality and poor growth performance [13,15,23]. Nevertheless, no difference was observed between the AUCs for the univariable models including either birth or weaning BW as predictors, indicating that either variable could be used to identify the pigs that would require more time to reach target slaughter weight. However, farmers could find the weaning BW cut-off value more valuable because most farms sort pigs by size or BW at weaning as a routine management practice. It is worth mentioning that the AUCs obtained in our study are considered less-moderate accurate [29]. This was likely due to the number of “controls” (i.e., animals not reaching target slaughter weight at 22 weeks of age) being only three more pigs than the number of “cases” (i.e., pigs reaching target slaughter weight at 22 weeks of age), when, ideally, the number of controls should be twice the number of cases. Moreover, it is likely that the less-moderate accurate AUCs observed in this study reflect the fact that birth and/or weaning body weight predict subsequent growth performance only to a certain extent, as growth performance is also influenced by other factors such as husbandry practices, nutritional strategies and animal health. Future studies are therefore required where other factors affecting reaching target slaughter weight in a timely manner are also included in the analyses.

We acknowledge that the cut-off values obtained in the present study using the regression tree and ROC curve analyses may not be extrapolated to other farms with a different production system, genetics, management strategies or sanitary status, as growth performance can be affected by other factors not presented in this study. Nevertheless, pig farmers could use regression tree and/or ROC curve analyses as decision-making tools to identify slow growing pigs earlier in life. The cut-off values identified could be used as a first indicator for pig producers to determine, from an economic standpoint, whether to cull low birth weight pigs or implement new management and nutritional strategies for slow growing pigs. As a result, two production flows could be created, always treating the slow growing pigs “off-site” from the normal production flow in an all-in-all-out production systems [53]. Re-grouping slow growing pigs does not improve their growth performance [18,54,55], unless different management and nutritional strategies are implemented, such as milk supplementation [2], cross-fostering [56], the development of high specifications diets post-weaning [57,58,59], increasing feeder space or establishing different phase feeding strategies during the grow-finisher period [60]. These strategies could improve the growth performance of slow growing pigs, leading them to partially catch up with their faster growing counterparts, maximizing financial returns.

## 5. Conclusions

This study provides a better understanding of the key performance indicators for grow-finisher pigs classified as small or big at birth and weaning based on their BW. Pigs that are born small and wean small have poorer lifetime growth performance and are not able to catch up with their heavier counterparts classified as big either at birth or at weaning. In addition, birth BW might not be the best predictor of subsequent growth performance, as some light birth BW pigs can show compensatory growth. Nevertheless, slow growing pigs had similar feed efficiency to pigs that were heavier at weaning. However, they spent more time in the facilities to reach target slaughter weight. This may lead to production inefficiencies. Pigs that were born heavier but were light at weaning showed compensatory growth during the grow-finisher period but had higher ADFI and FCR compared to the rest of the batch. Future research should focus on this group of pigs in economic terms and in relation to their carcass traits. The methods and cut-off values obtained for birth and weaning weight in this study may aid pig farmers as a decision-making tools to identify slow growing pigs early in life. As a result, pig farmers could design new management and/or nutritional strategies targeting slow growing pigs to improve their performance, thereby increasing production efficiency and farm profitability.

## Figures and Tables

**Table 1:** Descriptive statistics for birth body weight (BW), weaning BW, average daily gain (ADG) during lactation, sow parity and litter size [Mean ± Standard deviation (SD)] for pigs (n = 36 per group) classified according to their birth BW as SMALL (BW ≤ 1.15 kg) or BIG (BW > 1.15 kg), and re-classified according to their weaning BW as SMALL (BW ≤ 5.5 kg) or BIG (BW > 5.5 kg), yielding a 2 × 2 factorial arrangement.

Trait	SMALL–SMALL		SMALL–BIG		BIG–SMALL		BIG–BIG		*p*-Value
	Mean	SD	Mean	SD	Mean	SD	Mean	SD	
Birth BW, kg	0.9 ^b^	0.13	1.0 ^b^	0.13	1.4 ^a^	0.22	1.4 ^a^	0.19	<0.001
Weaning BW, kg	4.0 ^d^	0.95	6.8 ^b^	0.99	4.6 ^c^	0.74	8.0 ^a^	0.83	<0.001
ADG lactation, g	121.1 ^c^	39.00	228.3 ^b^	33.39	128.5 ^c^	29.43	257.3 ^a^	31.15	<0.001
Sow parity	3.7	2.01	3.0	2.17	4.0	2.04	3.9	2.09	0.189
Litter size	16.9	2.55	16.9	2.67	16.7	2.81	17.5	2.43	0.614

^a–b^ Within rows, significant differences between groups (*p* < 0.05).

**Table 2:** Body weight (BW), average daily gain (ADG), average daily feed intake (ADFI) and feed conversion ratio (FCR) from 6 to 20 weeks of age (Least square means [LS mean] ± Standard error mean [SEM]) for four groups of pigs classified according to their birth BW as SMALL (BW ≤ 1.15 kg) or BIG (BW > 1.15 kg), and re-classified according to their weaning BW as SMALL (BW ≤ 5.5 kg) or BIG (BW > 5.5 kg).

Trait	Birth BW × Weaning BW					*p*-Value		
	SMALL–SMALL	SMALL–BIG	BIG–SMALL	BIG–BIG	SEM	Birth BW	Weaning BW	Interaction
BW, kg								
6 wk	8.3 ^d^	9.9 ^c^	11.7 ^b^	12.9 ^a^	0.29	<0.001	<0.001	0.479
20 wk ^1^	86.1 ^b^	100.3 ^a^	100.6 ^a^	104.7 ^a^	2.22	<0.001	<0.001	0.025
ADG, g	868.4 ^b^	975.6 ^a^	944.1 ^a^	977.6 ^a^	21.02	0.001	0.066	0.081
ADFI, g	1690.1 ^b^	1948.7 ^a^	2133.2 ^a^	1999.5 ^a^	52.41	0.235	<0.001	<0.001
FCR	1.91 ^b^	1.96 ^b^	2.19 ^a^	2.00 ^b^	0.02	0.005	<0.001	<0.001

^1^ 20 weeks of age corresponds to the time when the first group of pigs reached 110 kg of BW and were sent to slaughter. ^a,b^ Within rows, significant differences between groups (*p* < 0.05).

**Table 3:** Average daily gain (ADG), average daily feed intake (ADFI), feed conversion ratio (FCR) and days to target slaughter weight (DTSW) (Least square means [LS mean] ± Standard error mean [SEM]) from six weeks of age until all pigs reached 110 kg of target slaughter weight for four groups of pigs classified according to their birth BW as SMALL (BW ≤ 1.15 kg) or BIG (BW > 1.15 kg), and re-classified according to their weaning BW as SMALL (BW ≤ 5.5 kg) or BIG (BW > 5.5 kg).

Trait	Birth BW × Weaning BW					*p*-Value		
	SMALL–SMALL	SMALL–BIG	BIG–SMALL	BIG–BIG	SEM	Birth BW	Weaning BW	Interaction
ADG, g	849.6 ^b^	939.7 ^a^	911.2 ^a^	937.7 ^a^	14.01	<0.001	0.035	0.025
ADFI, g	1787.7 ^b^	1906.1 ^b^	2051.1 ^a^	1884.9 ^b^	33.72	0.48	<0.001	<0.001
FCR	2.12 ^b^	2.04 ^b^	2.26 ^a^	2.01 ^b^	0.03	<0.001	0.073	0.015
DTSW, d	167.1 ^b^	153.0 ^a^	155.3 ^a^	150.4 ^a^	2.04	<0.001	<0.001	0.025

^a,b^ Within rows, significant differences between groups (*p* < 0.05).

**Table 4:** Performance [Area under the curve (AUC) and 95% confidence interval (CI)], *p*-value, sensitivity and specificity for the optimal cut-off value to identify pigs that would reach target slaughter weight [i.e., 110 kg of body weight (BW)] at 22 weeks of age considering birth BW, weaning BW and birth BW + weaning BW as predictor variables.

Predictor Variable	AUC, % (95% CI)	*p*-Value	Sensitivity, %	Specificity, %	Optimal Cut-Off Value, kg
Birth BW	72.7 (64.0–81.5)	<0.001	71.6	70.3	1.1
Weaning BW	68.4 (59.4–77.5)	0.001	77.6	53.1	6.7
Birth + Weaning BW	76.3 (67.8–84.8)	<0.001	-	-	-
